# Assessment of the trends in lipoprotein(a) concentration in high-risk cardiovascular patients: A retrospective study

**DOI:** 10.5339/qmj.2025.16

**Published:** 2025-03-05

**Authors:** Terry Gbaa, John Bolodeoku, Katherine Morris, Simon Whitehead

**Affiliations:** ^1^Lipid Clinic, Cardiology, Hampshire Hospitals NHS Foundation Trust, Basingstoke, UK; ^2^Biochemistry, Hampshire Hospitals NHS Foundation Trust, Basingstoke, UK *Correspondence: Terry Gbaa. Email: terrygbaa@yahoo.co.uk

**Keywords:** Lipoprotein(a), LPA, Lp(a), cardiovascular disease, ApoE, LDL-C

## Abstract

**Background:**

Cardiovascular disease (CVD) affects 500 million people globally, with mortality over 20 million. In the UK, the financial burden is estimated to be approximately £54 billion. Consequently, Lp(a) has been incorporated as an additional biomarker for cardiovascular risk stratification. It is used as a superior marker over the traditional marker LDL-C.

**Methods:**

This was a single-centre retrospective study conducted at Hampshire Hospital NHS Foundation Trust spanning 16 months (September 2022–January 2024). Lp(a) results were retrieved from the laboratory database and assessed for trends. The distribution of Lp(a) results was also compared with the values outlined in the HEART UK consensus statement (2019). Additionally, demographic characteristics such as age, sex, medical history, and lifestyle factors were collected. Personal details, including names, addresses, and phone numbers, were anonymised to ensure confidentiality. A total of 192 patients were included in the study. These patients were referred for a lipid panel by lipid specialists (172), GP surgeries (10), cardiologists (5), unspecified consultants (3) and endocrinologists (2). All patients were over 18 years of age. They were attending the clinic and had been screened for dyslipidaemia and high cardiovascular risk, including conditions such as familial hypercholesterolaemia, renal dysfunction, and those on antilipid therapy.

**Results:**

The demography included 99 (52%) females and 93 (48%) males. The chronological age (mean ± SD) was 61.17 ± 13.18 for females and 53.91 ± 12.84 for males (*p* < 0.001). Additionally, the Lp(a) values were 126.50 ± 118.92 and 135.33 ± 99.59 (*p* < 0.01) for females and males, respectively. The analysed samples were categorised as normal ( ≤ 32 nmol/L) and abnormal (>32 nmol/L) concentrations of Lp(a), with normal results observed in 104 patients and abnormal results in 88 patients: Lp(a) ≤ 32 nmol/L (54%) versus >32 nmol/L (46%), *p* < 0.0001. According to the CVD risk groupings established by HEART UK, 54%, 12%, 18%, 15% and 1% of the patients had Lp(a) values of 12.2 ± 7.5, 52.20 ± 16.42, 147.14 ± 36.64, 291.71 ± 62.49, and 471.50 ± 28.99 nmol/L, classified as normal, minor risk, moderate risk, high risk, and very high risk, respectively.

**Conclusion:**

This study provided evidence supporting the inclusion of Lp(a) as an extra component in lipid profile testing. Elevated levels of Lp(a) are associated with an increased risk of CVD, which may be more significant than the risk posed by LDL-C. Incorporating Lp(a) as a routine biomarker in real-world clinical practice would accurately stratify cardiovascular risk, particularly for patients with elevated Lp(a) concentrations, and could potentially be a more significant risk than LDL-C in individuals at high risk for CVD, especially for those ≥ 50 years of age.

## Introduction

Cardiovascular disease (CVD) is a chronic condition responsible for a large proportion of deaths worldwide. It is the leading cause of death, affecting over 500 million people globally. In 2021, CVD accounted for 20.5 million deaths.^1^ According to the British Heart Foundation, approximately 7.4 million people in the UK are living with CVD, with 6.4 million in England alone.^2^

The financial burden of CVD has been unprecedented, costing taxpayers around £54 billion to manage and maintain the plateau in the disease's prevalence.^3^ Data shows a consistent decline in CVD mortality rates from 1,049 cases per 100,000 individuals in the late 1960s to fewer than 300 cases per 100,000 in 2019.^4^ This progress reflects the advances made over the decades. However, to sustain this decline, it is essential to individualise and optimise drug treatments, with biochemical monitoring of lipid levels being of paramount importance.

Baseline lipid profiles have long been used to diagnose and assess the risk of CVD. According to research conducted by Hsu et al., changes in baseline lipid levels, particularly total cholesterol, triglycerides, and HDL-C, increase the risk of CVD.^5^ A fasting lipid profile is critical for risk stratification in individuals. However, some lipid particles, including Lp(a), are not routinely measured, which is now assessed by some specialised lipid clinics.

**Figure 1.**
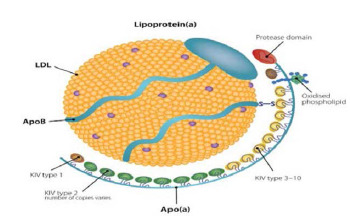
Lp(a) particle consisting of Apo(a), ApoB and LDL. Apo(a) surrounds the LDL particle with a protease domain and oxidised phospholipids, which include kringle types KIV type 1 and types 3–10.

Lp(a) has been identified as an independent risk factor for CVD and may be elevated even when LDL-C levels are normal. Similar to LDL-C, elevated Lp(a) levels have been associated with an increased risk of developing CVD.^6^ Lp(a) was discovered by Norwegian scientist Kåre Berg in the early 1960s.^7^ It is synthesized in the liver and consists of a polymorphic structure, with apolipoprotein(a) (Apo(a)) linked to the apolipoprotein B (ApoB) component of the LDL particle via a disulphide bond, as shown in Figure 1. Lp(a) is genetically determined, with its levels remaining stable throughout life, regardless of lifestyle factors. Additionally, the LPA gene is associated with ApoE.^8^

Lp(a) plays a physiological role in inflammation, wound healing, and cardiac remodelling. However, it is also susceptible to oxidative stress, leading to proinflammatory processes such as atherosclerosis. This oxidative damage can affect the layers of the cardiovascular vessels, particularly the intima-media.^9^

Early data suggest an association between Lp(a) levels and both atherosclerotic cardiovascular disease (ASCVD) and valvular aortic stenosis, although the exact mechanism is not entirely clear.^10^ Lp(a) testing may be beneficial for refining risk assessment in individuals with borderline cardiovascular risk. One study found that Lp(a) levels were highest in the Black population, showing a three-fold increase among younger and female patients globally. However, it is measured in only a small fraction of ASCVD patients.^11^

Therefore, this study aims to determine the trends of Lp(a) in high-risk CVD individuals and provide insights into its demographic and age stratifications at a lipid clinic of the Hampshire Hospital NHS Foundation Trust in the UK. This would provide an overview of how personalised therapy for patients with abnormal Lp(a) levels can be prioritised.

## Methods

### Study design and population

This was a single-centre study conducted at Hampshire Hospital NHS Foundation Trust. It used a retrospective design carried out over a 16-month period (September 2022–January 2024). Lp(a) results were retrieved from the laboratory database and assessed for trends. Furthermore, the distribution of Lp(a) results was compared with the values outlined in the HEART UK consensus statement (2019). Additionally, demographic characteristics such as age, sex, medical history, and lifestyle factors were retrieved. To ensure confidentiality, personal details, including names, addresses, and phone numbers, were anonymised.

A total of 192 patients were included in the study. These patients were referred for a lipid panel by lipid specialists (172), GP surgeries (10), cardiologists (5), unspecified consultants (3), and endocrinologists (2). All patients were aged 18 years or older. They were attending the clinic and had been screened for dyslipidaemia and high cardiovascular risk factors, including conditions such as familial hypercholesterolaemia, renal dysfunction, and those on antilipid therapy.

### Laboratory analysis

Fasting blood samples were collected in 5 ml vacutainer tubes. These samples were separated using a tabletop centrifuge at 3,000 rpm for 10 minutes. The plasma was pipetted into cryovials. Samples that were not analysed immediately were stored at -20°C. The samples were analysed for Lp(a) using an automated chemistry analyser on the Roche Cobas e411 (Beckman Coulter, CA, USA).

### Statistical analysis

The Shapiro-Wilk test was used to assess the normality of data distribution. For Gaussian data, the Student's t-test and one-way analysis of variance (ANOVA) were used. The Mann-Whitney U test was used for non-Gaussian data. Results were presented as mean ± standard deviation (SD) and median, while categorical variables are presented as numbers or percentages. We used the Student's t-test and Mann-Whitney U to determine two independent means, as well as one-way ANOVA and tests to determine more than three independent sample means.

Furthermore, we used the interquartile range (IQR) for datasets that were skewed, which were represented by box and whisker plots. Statistical significance was defined as *p* < 0.05, while *p* < 0.01 indicated a high level of statistical significance.

### Ethical considerations

Patients were informed about the use of their data, and their informed consent was obtained. Sensitive data were anonymised to ensure privacy and data protection under the General Data Protection Regulation standards. Patient details were stored in a secure cloud environment, using a key chain system to prevent the risk of data breaches.

## Results

The demography included 99 (52%) females and 93 (48%) males. The mean chronological age was 61.17 ± 13.18 years for females and 53.91 ±  12.84 years for males (*p* < 0.001). Additionally, the Lp(a) values were 126.50 ± 118.92 nmol/L for females and 135.33 ± 99.59 nmol/L (*p* < 0.01) for males, as shown in Table 1.

**Figure 2.**
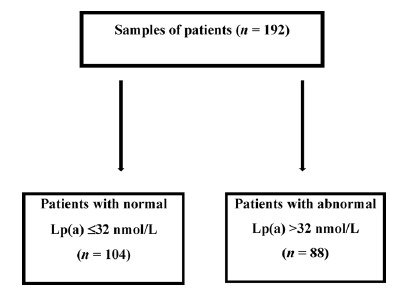
A flowchart of the Lp(a) values for each group of patients.

Figure 2 shows a flowchart categorising patients based on their Lp(a) values, distinguishing between normal levels ( ≤ 32 nmol/L) and abnormal levels (>32 nmol/L). The analysis of the collected samples revealed that 104 patients exhibited normal Lp(a) concentrations ( ≤ 32 nmol/L), while 88 patients were categorised as having abnormal Lp(a) concentrations (>32 nmol/L).

**Figure 3.**
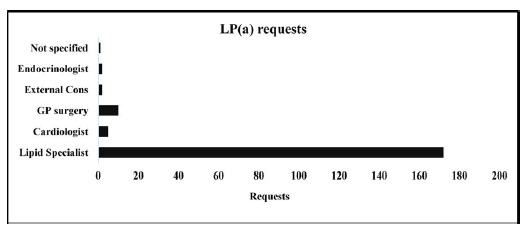
Lp(a) requests and requestors (n = 192)

Lipid specialists represented the largest group of requestors, followed by GP surgery, and the lowest category of requestors was not identified as there were no specific descriptions of requestors (Figure 3).

**Figure 4.**
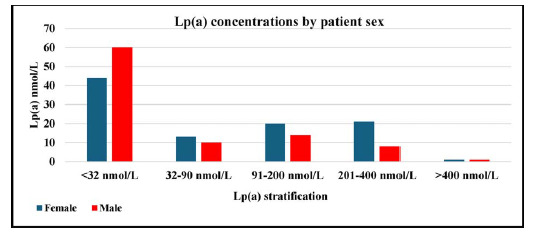
Lp(a) values stratified by sex (n = 192)

The group with Lp(a) values ≤ 32 nmol/L comprised a higher number of males (60) compared to females (44), in contrast to the group with elevated Lp(a) values (>400 nmol/L), which displayed an equal distribution of males and females (Figure 4).

The cut-off range for Lp(a) between 32 and 90 nmol/L showed statistical significance for both male and female groups, while other categories showed no statistical significance, as detailed in Table 2.

**Figure 5.**
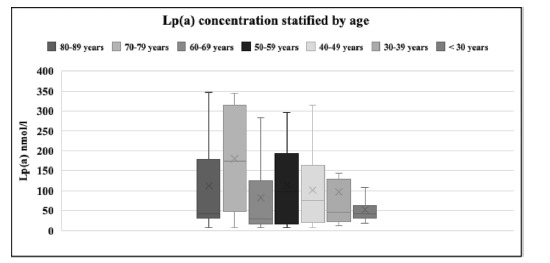
Stratification of Lp(a) by age. < 30 years: 35 nmol/L (IQR 26.5–72); 30–39 years: 38 nmol/L (IQR 22–113.5); 40–49 years: 58 nmol/L (19.5–169.5); 50–59 years: 91.5 nmol/L (IQR 15.7–194); 60–69 years: 29 nmol/L (IQR 16–125); 70–79 years: 185.5 nmol/L (IQR 48.5–315); 80–89 years: 39 nmol/L (IQR 29–237).

The age group under 30 years showed the lowest mean Lp(a) value of 54 ± 48.40 nmol/L (minimum 35 nmol/L and maximum 109 nmol/L), while the age group of 70–79 years presented the highest mean value of 184.20 ± 132.00 nmol/L (minimum 8 nmol/L and maximum 342 nmol/L). Additionally, Figure 5 showed stratification of Lp(a) by age, showing that the median values of Lp(a) were 29 nmol/L (lowest) for the age group of 60–69 years and 185.5 nmol/L (highest) for the age group of 70–79 years.

**Figure 6.**
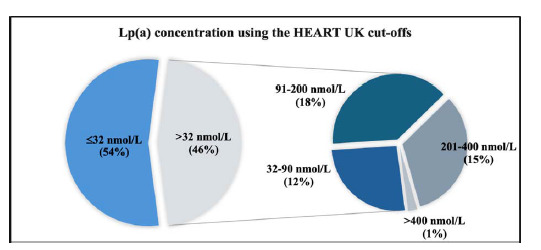
The HEART UK cut-off for elevated levels of Lp(a). The pie chart represents both the normal and abnormal results of Lp(a), highlighting the different risk categories associated with the elevated Lp(a) levels.

Figure 6 presents a pie chart illustrating the distribution of Lp(a) concentrations, with 54% of individuals having levels ≤ 32 nmol/L, while 46% exhibiting levels >32 nmol/L (*p* < 0.0001). Among the 46% of individuals with elevated Lp(a) levels (>32 nmol/L), further analysis using the HEART UK cut-off revealed that the highest proportion of patients fell in the 91–200 nmol/L range (18%), classified as moderate risk, while the lowest proportion fell in the >400 nmol/L range (1%), classified as very high risk.

Table 3 presents a risk stratification that illustrates the cut-off for HEART UK, with 104 patients within the reference range < 32 nmol/L, 29 participants classified as high risk, and only 2 participants classified as very high risk at 201–400 nmol/L and >400 nmol/L, respectively. Using the CVD risk groupings associated with HEART UK, it was observed that 54%, 12%, 18%, 15%, and 1% of the patients had Lp(a) values of 12.2 ± 7.5, 52.20 ± 16.42, 147.14 ±  36.64, 291.71 ± 62.49, and 471.50 ± 28.99, which were classified as normal, minor risk, moderate risk, high risk, and very high risk, respectively.

Table 4 shows the demographic stratification of elevated Lp(a) levels, with moderate risk males versus females (7% vs. 10%) and high-risk males versus females (4% vs. 11%). However, no significant difference was found between males and females in the very high-risk group.

## Discussion

The risk of CVD has long been associated with elevated lipid levels, particularly LDL-C, as well as total cholesterol and triglycerides. Recent evidence has highlighted an association between increased CVD risk and elevated Lp(a) levels. Lp(a) has a structure similar to LDL-C, and both are considered atherogenic. However, evidence suggests that Lp(a) may pose a higher cardiovascular risk than LDL-C.^12^ Additionally, the deposition of calcium in the arteries contributes to the development of coronary heart disease.^13^

In this study, we found that 46% of the patients had elevated Lp(a) concentrations, categorised into the ranges of 32–90, 91–200, 201–400, and >400 nmol/L, with 12%, 18%, 15%, and 1% of the patients falling into these categories, respectively. We also observed a statistically significant difference in mean Lp(a) levels between males and females (*p* < 0.01), with males having higher levels. This finding contrasts with a study indicating that women with type 2 diabetes had higher Lp(a) levels than men, although the difference was not statistically significant.^14^

Furthermore, our study revealed that within the individual categories defined by the HEART UK Lp(a) classification, a greater proportion of females than males had elevated Lp(a) levels in the ranges of 32–90 nmol/L (8% vs. 5%), 91–200 nmol/L (10% vs. 7%), and 201–400 nmol/L (11% vs. 4%). Conversely, a higher proportion of males fell within the normal Lp(a) range compared to females (31% vs. 23%).

Age significantly influenced Lp(a) levels. As expected, individuals under 30 years of age had the lowest Lp(a) levels, while those in the 70–79 age group exhibited the highest Lp(a) levels, indicating an elevated risk of CVD and other comorbidities, such as cerebrovascular accidents. A study conducted in Copenhagen demonstrated a similar trend, showing a gradual increase in Lp(a) concentrations in women over 50 years compared to men of the same age.^15^ In our study, the interquartile ranges for the lowest and highest Lp(a) levels were 29 nmol/L (IQR 16–125) for those aged 60–69 years and 185.5 nmol/L (IQR 16–315) for those aged 70–79 years. Interestingly, the median Lp(a) levels gradually increased across all seven age groups, decreased in the 60–69 age group, reached a peak in the 70–79 age group, and then declined again in the 80–89 age group. The reason for this phenomenon remains unclear.

Additionally, we found that menopausal women had higher Lp(a) levels compared to males in the same age group. This trend was particularly evident in patients aged over 50 years, with Lp(a) levels reaching a peak between the ages of 61 and 70 years, followed by a subsequent decline. A study conducted by Aljawini et al. in Saudi Arabia found that postmenopausal women (57.1%) had higher Lp(a) concentrations than premenopausal women (19%).^16^ This may be due to the reduction in oestrogen levels, which plays an important role in inhibiting the transcription of the LPA gene.

In this study, we observed that only lipid specialists were requesting Lp(a) measurements, even though a routine fasting lipid profile typically does not include Lp(a) testing. Over the years, Lp(a) has shown a positive correlation with CVD in numerous outcome datasets. Therefore, it is crucial for both primary and secondary healthcare departments to recognise Lp(a) as a significant risk factor for CVD, potentially surpassing the risk posed by LDL-C alone.

The current National Institute for Health and Care Excellence (NICE) treatment guidelines do not include Lp(a) as a target for cardiovascular risk reduction. Standard lipid-lowering therapies, such as fibrates, statins, and ezetimibe, may not lower Lp(a) levels or reduce the overall risk of CVD. In fact, they may paradoxically increase Lp(a) concentrations.^17^ Lp(a) is associated with major adverse cardiovascular events, but current lipid-lowering drugs have limited effectiveness in reducing Lp(a) levels, likely due to its genetic regulation. At present, there is no definitive therapy for Lp(a) reduction, although several clinical trials are underway.^17–19^

New therapies in various stages of clinical trials, such as olpasiran, pelacarsen, and SLN360, work by inhibiting mRNA transcription. Studies have shown that these novel treatments reduce Lp(a) levels by 35–80%, 75–90%, and 46–98%, respectively, using antisense oligonucleotides. Current PCSK9 inhibitors have demonstrated a reduction in Lp(a) levels ranging from 19% to 27%, although this reduction is less effective. Additionally, studies have noted a reduction in HDL-C levels alongside Lp(a).^20^ A study by Sahebkar and Watts showed that PCSK9 inhibitors decreased Lp(a) by 30%.^21^ The future is promising for the development of more lipid-lowering drugs targeting Lp(a), particularly siRNA-based oligonucleotide therapies.

## Conclusion

This study highlights the importance of considering Lp(a) as an additional analyte in lipid profile assays. Incorporating Lp(a) as a routine biomarker in clinical practice could more accurately stratify cardiovascular risk, particularly for patients with elevated Lp(a) concentrations. In fact, Lp(a) may pose a greater risk factor than LDL-C in individuals at high risk of CVD, especially those aged 50 years and older. Assessing Lp(a) levels could fill a critical gap in effectively triaging patients into low- or high-risk groups, helping to optimise their antilipid therapy based on cardiovascular risk, age, and associated comorbidities. Routine Lp(a) evaluation may also lead to the redefinition of current guidelines and policies, ultimately reducing the overall burden of CVD.

### Limitations

This study did not consider race as a variable, despite evidence suggesting that the Black ethnic population tends to have higher Lp(a) levels than other ethnic groups. This aspect will be explored in future studies. Additionally, the study was conducted at a single centre and would have benefitted from multicentre collaboration, but logistical limitations precluded this possibility. Expanding to multiple centres will be considered in future research.

### Competing interests

The authors have no conflicts of interest to declare.

## Figures and Tables

**Table 1.** Demographic distribution for Lp(a) and age (n = 192).

	Lp(a), mean ± SD (nmol/L)	Age, mean ± SD (years)

Female	126.50 ± 118.92 Min 7.00, Max 492	53.91 ± 12.84

Male	135.33 ± 99.59 Min 7.00, Max 451	61.17 ± 13.18

*P*	< 0.01	< 0.001


Statistically significant values are indicated at *p* < 0.05. The Student's t-test was used to compare the mean levels of Lp(a), while the Mann-Whitney U test was used to compare the differences in mean ages between males and females.

**Table 2.** Comparison of male versus female HEART UK Lp(a) cut-off for each category (n=192)

HEART UK cut-off	Male Lp(a) mean ± SD (nmol/L)	Female Lp(a) mean ± SD (nmol/L)	*p*

≤ 32 nmol/L	17.03 ± 8.01	16.29 ± 7.53	0.350

32–90 nmol/L	40.40 ± 16.86	49.62 ± 15.23	< 0.05

91–200 nmol/L	144.36 ± 362.53	158.00 ± 33.95	0.120

201–400 nmol/L	259.25 ± 48.64	291.71 ± 62.49	0.100

>400 nmol/L	471.50 ± 28.99	471.50 ± 28.99	1.000


Statistically significant values are indicated at *p* < 0.05. The Student's t-test was used to compare the mean cut-off values of Lp(a) for each group.

**Table 3.** Risk stratification using the HEART UK cut-off (n=192)

No.	Risk	HEART UK cut-off (nmol/L)	Lp(a), mean ± SD (nmol/L)

104	Normal	< 32	12.2 ± 7.50

23	Minor	32–90	52.20 ± 16.42

34	Moderate	91–200	147.14 ± 36.64

29	High	201–400	291.71 ± 62.49

2	Very high	>400	471.50 ± 28.99

192		*p*	< 0.0001


Statistically significant values are indicated at *p* < 0.05. A one-way ANOVA was used to compare the difference in Lp(a) levels across each risk group.

**Table 4.** Demographic risk stratification using the Lp(a) HEART UK cut-off.

HEART UK cut-off (nmol/L)	Male (%)	Female (%)	Total (%)

< 32	60 (31)	44 (23)	104 (54)

32–90	10 (5)	13 (8)	23 (12)

91–200	14 (7)	20 (10)	34 (18)

201–400	8 (4)	21 (11)	29 (15)

>400	1 (0.5)	1 (0.5)	2 (1)

